# Poly(itaconic Acid) Hydrogels with Dynamic Covalent and Supramolecular Cross-Links for High and Tunable Mechanical Properties

**DOI:** 10.1021/acsapm.6c01440

**Published:** 2026-07-22

**Authors:** Henri Savolainen, Maximilian J. L. Hagemann, Mika Salmi, Jouni Partanen, Mario Piedrahita-Bello, Olli Ikkala

**Affiliations:** † Department of Applied Physics, 174277 Aalto University, Espoo 02150, Finland; ‡ Department of Energy and Mechanical Engineering, Aalto University, Espoo 02150, Finland

**Keywords:** hydrogels, Sustainable polymers, Supramolecular bonds, Dynamic covalent bonds, superabsorbents

## Abstract

Aiming toward sustainability, poly­(itaconic acid) has been considered as an alternative to the widely used fossil-based poly­(acrylic acid). Its application has been, however, limited by its lower mechanical properties due to challenges in the polymerization processes. Herein, we report photopolymerizable and thermoreversible hydrogels based on poly­(itaconic acid) allowing tunability from high strength (∼1000 kPa) plastically yielding materials to soft elastomeric-like stretchability, also allowing thermoresponsive flow, and a relatively high water absorption of 384 g/g. The thermal gel melting stems from the dynamic covalent bonds, in combination with metal-ion-mediated supramolecular connections. The materials facilitate on-demand water stability while allowing gel-to-sol melting for thermal extrusion for 3D-printing, a combination not observed in classic permanent covalent networks. To promote sustainability, biosourceable starting materials are used without additional solvents or purification steps. Given the biocompatibility of the components, potential applications can be foreseen, such as tissue growth scaffolds, medical and hygienic superabsorbents, and tough hydrogels for soft robotics.

## Graphical abstract

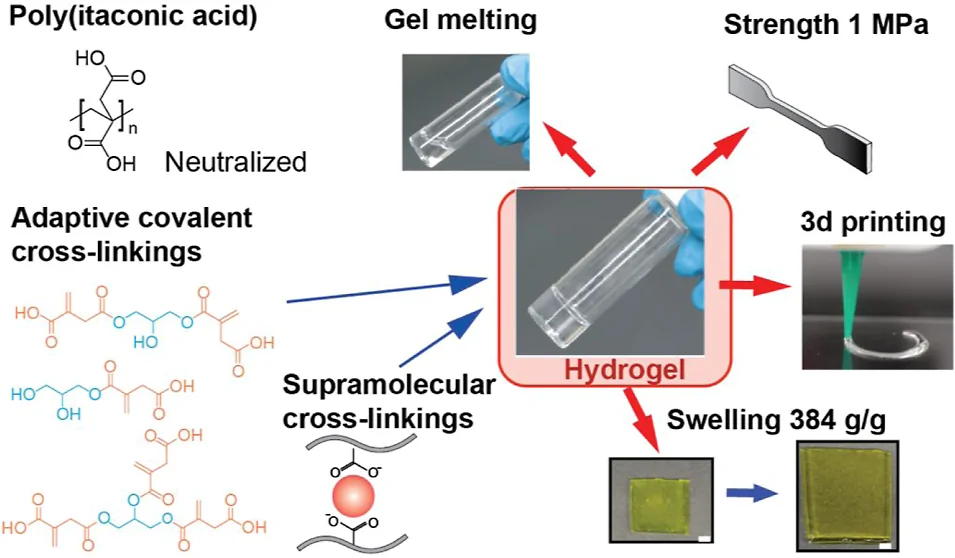

## Introduction

Fossil-based polymers, such as acrylates, have dominated technical applications due to their ease of processing, low cost, and high chemical versatility. However, their continued usage worsens the global issues of emerging plastic waste and microplastic accumulation, thus calling for new approaches.^1,2^ In this regard, polymers based on biosourced, renewable, and biodegradable monomers have been attractive but have often required cumbersome, costly, and energy-intensive processing and manufacturing compared to the already established fossil-based materials. This has limited their application potential, making them often unscalable.^3^ Among the most important fossil-based polymers, poly­(acrylic acid) (PAA) has been particularly relevant due to its versatility, e.g., being a dispersing, suspending, thickening, and emulsifying agent in pharmaceutical, cosmetic, and paint applications, in water purification, and providing superabsorption in hygiene applications,^4^ totaling an annual production of ca. 6 million metric tons in 2021.^5^

Therefore, identification of sustainable alternatives for PAA is particularly relevant. One way of tackling this challenge is the utilization of poly­(itaconic acid) (PIA), which is biosourceable and enzymatically biodegradable (for example by α-amylase or soil buried testing)^4,6^ and where the IA monomer is significantly less-toxic^7^ compared to acrylic acid (AA).^8^ Unlike AA, IA can be biochemically produced by fermentation of biomass using lignin and the fungus *Aspergillus terreus*.^9^ Being obtainable from softwood as a resource, IA-based polymers have a lower environmental impact in comparison to their oil-based acrylic counterparts.^10^ Initial steps have already been taken therein, e.g., IA has been used in the fabrication of prepolymer resins to substitute AA^11^ and explored as a component for a biobased 3D printable material.^12^ Recent market analyses show that, as of 2026, the cost of the AA monomer is comparable to that of the IA monomer.^42,43^

Still, transforming PIA into useful polymeric materials involves considerable challenges, especially those related to achieving high mechanical properties.^13^ This mainly stems from the limited molecular weight of PIA-based linear polymers allowed by state-of-the-art polymerizations, which promote material brittleness.^14^

In order to address these issues to allow promoted degrees of polymerizations for improved mechanical properties, we explore aqueous network architectures, i.e., hydrogels, in combination with covalent and supramolecular cross-links. In general, hydrogel networks can allow significantly enhanced strengths by using tailored permanent covalent cross-linkers such as *N*,*N*-methylenebis­(acrylamide) (BIS). However, permanent covalent cross-linking hinders the recycling and degradation of the polymer, as well as limits any further processing or reprocessing.^15^ Another approach to solve this issue could be to use dynamic covalent or supramolecular networks to reduce brittleness and to improve stretchability while retaining recyclability, such as vitrimers.^16,17^

In this work, a synthetic route for stretchable, malleable, and meltable PIA-based hydrogels is introduced to tackle these issues. Synthesis is done via photopolymerization of the gel phase, employing covalent dynamic cross-links to allow exchangeable bonds in combination with supramolecular bonds. Such a synergistic approach not only showed enhanced physical properties but also allowed their fine-tuning for targeted applications. Therein, we first describe the synthesis of a cross-linker derived via a fully biosourced route by IA and glycerol, i.e., two materials obtainable via fermentation and saponification, respectively.^18^ No purifications (including filtrations or postsynthetic treatments) or organic solvents are used throughout the whole present synthetic process.^19^

Photoinitiated radical polymerization is considered more sustainable than thermally initiated radical polymerization as the energy requirements of using light as an initiator are generally seen as lower.^20^ Therein, even the photoinitiator can be selected to be biosourced, i.e., α-ketoglutaric acid. As an alternative polymerization approach, thermal initiation of itaconic acid has been extensively studied.^14,21^ It has been concluded that typical thermal radical initiators such as ammonium persulfate are not suitable for polymerizing itaconic acid due to increased production of nondesired side products. Also step degradation of the initiator takes place due to the carboxylic groups of itaconates, inducing only a short initiation step unfavorable for the polymerization due to possible resonance leading toward very low molecular weight of the chain.

This work explores also the important role of different metal ions in the supramolecular polymer networks related to their dynamics, as well as plasticization through further glycerol addition. By tuning the composition, we can control/tune whether the hydrogel is mechanically strong, soft, and 3D-printable, or superabsorbing. This degree of tunability thus suggests application tailoring for emerging fields such as wound sealing, adhesives, cell culture, scaffolding, superabsorbents, and tough hydrogels for soft robotics.^22,23^ We emphasize that an exclusively covalent cross-linked hydrogel does not allow melting and thus 3D printing. As a further advantage toward the acrylic counterpart, this approach also avoids the need for postprocessing curing. In the following section, the mechanical and physical properties, of the hydrogels at different compositions are described.

The main difference of IA in comparison to AA is the presence of two carboxylic acids with different p*K*_a_ values (Figure 1a). This allows wider possibilities for modification than AA and results in an abundance of free carboxylates in the PIA. These moieties thus play a crucial role in the dynamic covalent connections in our material by catalyzing transesterification, allowing heat-triggered melting at slightly elevated temperatures, which we could exploit to print hydrogels via thermal extrusion. To improve the aqueous solubility during polymerization and thus suppress aggregation and precipitation of the monomers, equal molar amounts of IA (1.0 equiv) and NaOH (1.0 equiv) were added. This resulted in a nominal neutralization degree of 50% and was used to prevent low-molecular weight and brittleness of resulting polymers.^14^

**Figure 1:**
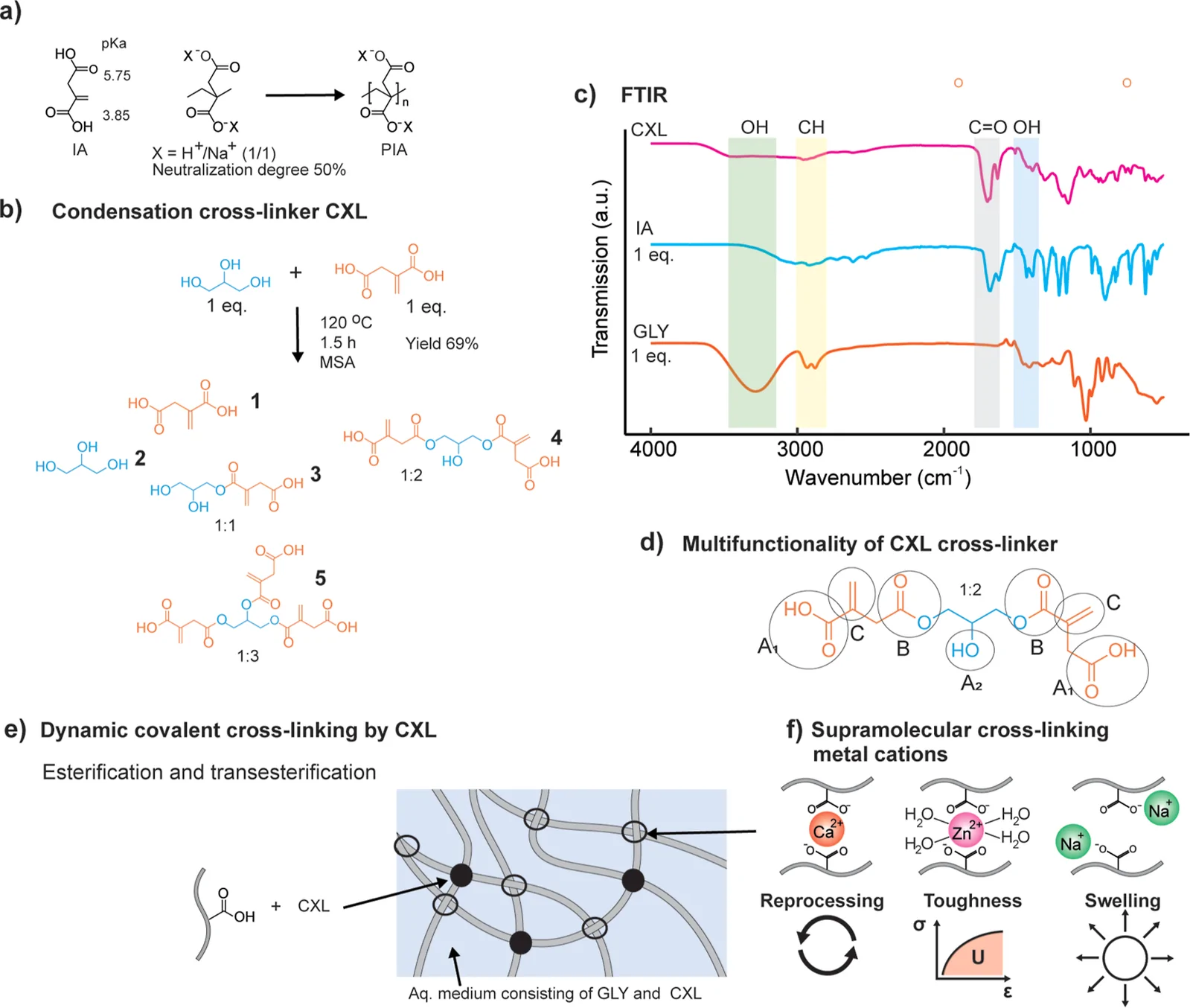
Schematics for the photopolymerized polyitaconic acid hydrogels based on the combination of dynamic covalent and metal-ion supramolecular cross-linking. (a) Chemical structures of IA, 50% neutralized IA, and PIA. (b) Methanesulfonic acid (MSA)-catalyzed chemical condensation between itaconic acid and GLY without using added solvents, leading to condensation cross-link mixture CXL. The several reaction products contribute to the dynamic covalent network formation (see also Figure S1). (c) FTIR spectra demonstrating chemical reaction between IA and GLY. (d) CXL are multifunctional, as illustrated by one of the molecular species: The carboxylic acid (A_1_) and alcohol (A_2_) sites are involved in esterification and transesterification, while A_1_ also functions as a potential binding site for ion bindings; B: Ester-groups functioning as a dynamic covalent cross-linker; and C: Vinyl bonds for possible postmodifications by photopolymerization. (e) Schematics for the dynamic covalent bond with CXL based on the –COOH functionalities of the partially neutralized polymer. (f) Schematics for the metal-ion-based supramolecular cross-links with CXL based on the carboxylate functionalities of the partially neutralized polymer. (a) PIA: The weight fraction of poly­(itaconic acid) (PIA) in the final polymerized hydrogel (wt %) based on the initial half-neutralized IA aq. monomer concentration. (b) CXL/IA: The weight fraction of CXL cross-linker vs weight of IA monomers (wt %) which determines the density of the dynamic covalent cross-links. (c) GLY: The concentration of the added glycerol in the total aqueous mixture (wt %) which determines the level of plasticization. (d) Ca^2+^/IA or Zn^2+^/IA: The molar fraction of metal cations vs IA monomer (mol %) which determines the density of supramolecular cross-linking.

The importance of dynamic and dual cross-linking to tune gel properties has already been emphasized.^24^ Herein, dynamic cross-linkage was achieved with a novel condensate cross-linker (denoted CXL) to allow dynamic ester bonds. (a) The cross-linker is composed of the biosourceable components glycerol (GLY) and IA by their esterification (Figure 1b). Due to the multifunctionality of IA, several esterification architectures are possible whereof some representative ones are shown in Figure 1b (see also, Figure S1). (b) No purification steps are involved in the CXL synthesis by sustainable principles. (c) No additional solvents are used throughout the synthesis with glycerol functioning as a solvent and reactant. (d) All additionally utilized compounds, i.e., the condensation catalyst methanesulfonic acid (MSA) and photoinitiator 2-oxoglutaric acid for the IA polymerization, can be biologically sourced and are biodegradable.^4,25^

Note that common photoinitiators such as benzophenone or Irgacure 2959 did not result in the hydrogelation of the present compositions under UV irradiation for the same irradiation conditions. On the other hand, using thermally initiated radical polymerization with ammonium persulfate and tetramethylenediamine resulted in polymerization when heated at 60 °C overnight but did not lead to thermoreversible hydrogels and 3D-printability and were thus abandoned herein.^14,26^

## Materials and Methods

To synthesize the CXL cross-linker, IA (3.0 equiv) and GLY (1.0 equiv) were first stirred at 170 °C to ensure homogeneous mixing. The liquid mixture was subsequently cooled to 120 °C to add the condensation catalyst methyl sulfonic acid (MSA). Additionally, hydroquinone was added to inhibit the reaction of the vinyl groups. Conversion of the IA carboxylic acids by GLY was confirmed by FTIR spectroscopy (Figure 1c). This can be observed via the suppressions of the OH-stretching of GLY near 3300 cm^–1^ (green) and C–H stretching of GLY near 2850–3000 cm^–1^ (yellow) in the reaction product. Also, significant reduction of the hydroxyl absorption of the carboxylic acid group of IA around 1566 cm^–1^ (blue) and the shift of the carbonyl peak around 1700 cm^–1^ (gray) hints to a successful ester condensation for the product. Further confirmation for the successful ester condensation of GLY and IA was provided via 1H NMR spectroscopy (Figures S2–S4), where the spectra were explored in D_2_O and DMSO-*d*_6_. Therein, a downfield shift of the alkene protons upon esterification of IA was observed, in agreement with the literature,^11^ qualitatively suggesting that conversion takes place. To obtain more quantitative information on the conversion, the average integrals of the IA peaks at 5.72/6.12 ppm and 5.89/6.37 ppm for DMSO-*d*_6_ and D_2_O were compared with the slightly downfield shifted peak associated with the ester ones (Figures S2–S4). Therein, carboxylic acid conversion of around 31% was estimated. Due to diacidity of IA, several esterification architectures are formed as shown by ESI-QTOF spectroscopy (Figure S4).

As already pointed out, no purification of CXL was incorporated, as the unreacted residual IA and GLY appear in future steps as monomers and plasticizers, respectively. Hereby, the GLY can interact through hydrogen bonding with the carboxylic acid and carboxylate groups and plasticize the gel to promote stretchability while also allowing the formation of new ester bonds upon thermal treatment of the material.^27^ In this regard, the CXL cross-linker molecules are multifunctional (Figure 1d).

For the reaction mixture, an aqueous solution of the Na^+^-half-neutralized IA (Figure 1a) and cross-linker mixture CXL (Figure 1b) were mixed and photopolymerized to obtain a covalent ester hydrogel network (schematically shown in Figure 1e). Even if these hydrogels showed superior physical properties in comparison to the non-cross-linked ones, they also showed undesirable brittleness. To counteract, further GLY was added prior to polymerization as a plasticizer. In the case of metal addition, salts (CaCl_2_, ZnCl_2_, and NaCl) were added to this mixture (see the Supporting Information).

The hydrogels are multicomponent and involve complex behavior due to their compositions. We specified the compositions as follows:

All of the prepared compositions are listed in Table S1. As an example of the composition, the subsequent findings will show that the composition PIA 24 wt %, CXL/IA 4 wt %, GLY 42 wt %, and Ca^2+^/IA 0.5 mol % show a particularly useful balance for good mechanical properties, thermally driven gel melting to allow 3D-printing, and swelling.

## Results and Discussion

We first qualitatively show that the hydrogel mechanical properties can be widely tuned. Figure 2a shows the tensile properties upon a fixed covalent cross-linker CXL to IA ratio (CXL/IA 4 wt %) and a fixed supramolecular cross-linker ratio to IA (Ca^2+^/IA 0.5 mol %). Using a high PIA fraction of 42 wt % without a GLY plasticizer leads to relatively strong gels with a strength of nearly 600 kPa but also more brittle behavior (blue curve). Incorporation of a GLY plasticizer (27 wt %) and reducing the weight fraction of PIA to 30 wt % allows promoted ductility and evidence of yielding (green curve). A still higher fraction of GLY (42 wt %) and a lower fraction of PIA (24 wt %) lead to stretchable behavior and evidence of strain hardening (red curve).

**Figure 2:**
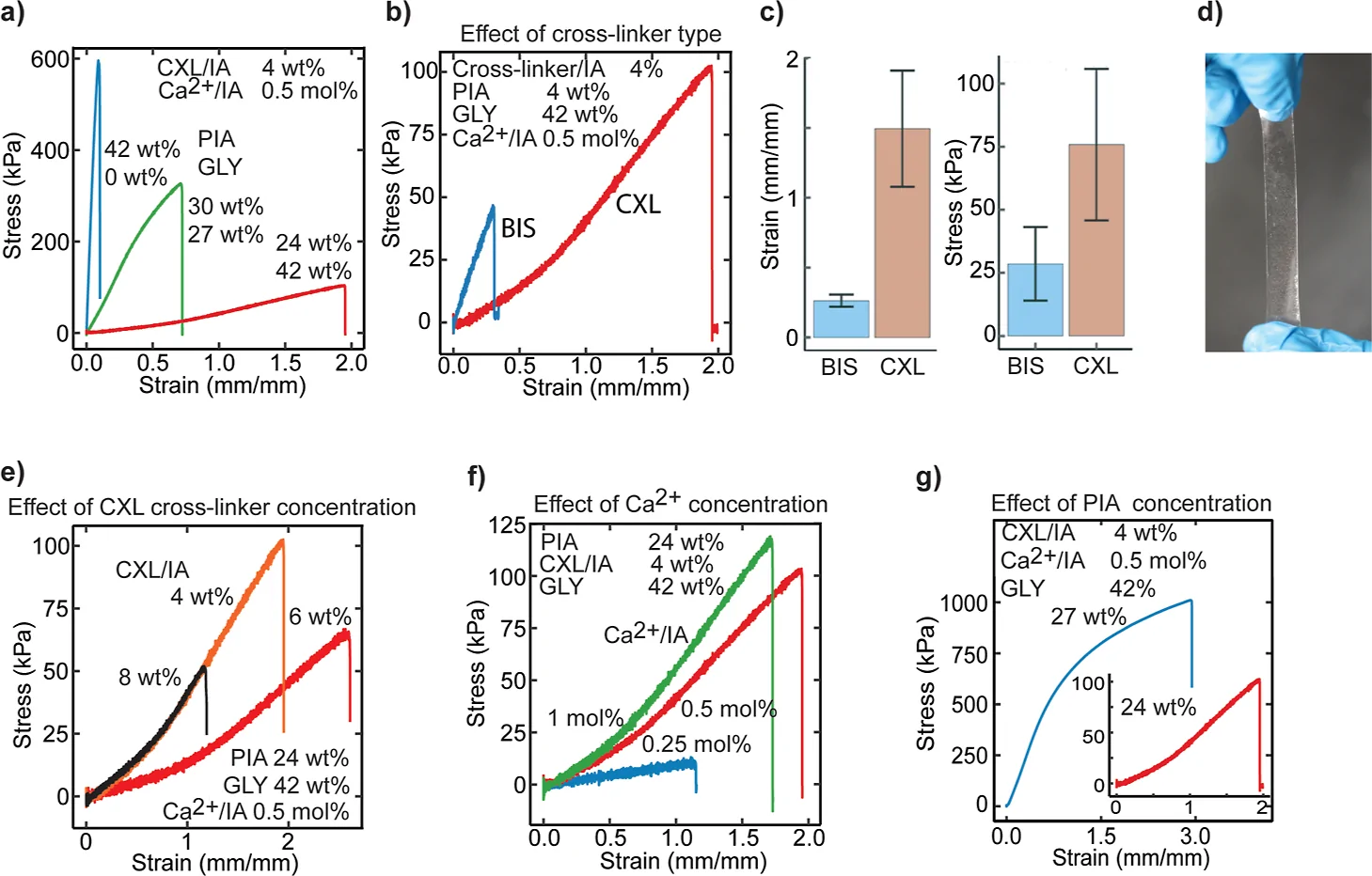
Mechanical properties of the hydrogels based on PIA with Na^+^-based neutralization degree 50% based on the dynamic covalent cross-linking by CXL and Ca^2+^-cation supramolecular cross-linking and additional glycerol plasticizer (GLY). The compositions are indicated in the panels. (a) Demonstration that proper selections of PIA and glycerol concentrations allow tuning of the hydrogel mechanical properties from brittleness to ductility until elastomer-like stretchability. (b,c) CXL allows ductility (PIA 24 wt %, CXL/IA 4 wt %, GLY 42 wt %, and Ca^2+^/IA 0.5 mol %) whereas the classic BIS cross-linker leads to brittleness. (d) Visualization of the stretchability of the CXL-based hydrogel of composition of (b,c). (e) Stress–strain curves of materials with different CXL/IA ratios. (f) Addition of supramolecular cross-linker has a pronounced effect to increase the mechanical properties. (g) A small increase in the concentration of PIA from 24 to 27 wt % leads to a high gel strength of ∼1000 kPa. At least *n* = 3, number of independently synthesized samples, have been measured.

To further show the potential of our approach with a dynamic covalent cross-linker, we compared the performance of CXL with the widely used acrylate cross-linker BIS (Figure 2b). A photograph of such a transparent stretchable CXL-based sample is shown in Figure 2d.

Our material not only presents a higher stretchability, it also outperforms BIS (Figure 2c). A factor for the poor mechanical performance of using BIS could be the significantly higher reaction rates in acrylates to itaconates.^28^ Due to the different reaction rates, in homogeneities are formed, causing nonuniform cross-linking whereas the itaconate-based cross-linkers match the reaction rate of the monomer, leading thus to a more uniform cross-linking density distribution.^28^ We see the effect also in the higher cross-linking of BIS in 15.7 ± 0.8 compared to 6.9 ± 0.3 mol/cm^–3^. Cross-linking density was derived using mechanical data from the theory of rubber elasticity.^29^

In Figure 2e, we demonstrate the effect of the weight fraction of the cross-linker CXL on the tensile properties. For this investigation, the PIA, GLY, and Ca^2+^ concentrations were kept constant (PIA 24 wt %, GLY 42 wt %, and Ca^2+^/IA 0.5 mol %). If no CXL was used, the hydrogel was brittle and waxy, not allowing for reliable tensile testing. Increasing CXL to CXL/IA 4 wt % leads to elastomeric-like behavior with a stress of 75.8 ± 30.0 kPa and strain of 1.5 ± 0.4 mm/mm (orange curve), while using 8 wt % leads to no further increase of mechanical properties. Increasing CXL/IA even higher shows processing difficulties due to increased viscosity. Typically, effective cross-link density is estimated via tensile tests.^29^ Therefore, we estimated and compared the effective cross-link density for different cross-linker concentrations and obtained values of 2.5 ± 0.7, 6.9 ± 0.4, and 4.5 0.8 mol/cm^–3^. Therefore, there exists an optimum concentration of CXL. We also note that samples with <4 wt % CXL are not stable in water and will dissolve over time (Figure S7).

As there exists free carboxylate sites after the initial neutralization, further Ca^2+^ ions were introduced by adding CaCl_2_ to the reaction mixture to induce supramolecular ionic bridging between the carboxylate groups to explore the mechanical properties of the hydrogel, as has been suggested in the recent literature for other materials.^30–32^ Addition of Ca^2+^ had a pronounced effect on the tensile properties, emphasizing the importance of the additional supramolecular cross-linking beyond the covalent cross-links. In Figure 2f, the PIA, CXL, and GLY concentrations are kept constant (PIA 24 wt %, CXL/IA 4 wt %, GLY 42 wt %) and the Ca^2+^ concentration is varied. In the absence of Ca^2+^, the hydrogels are brittle, not allowing proper tensile tests. Upon increasing Ca^2+^ concentration to Ca^2+^/IA 0.5 mol % leads to elastomer-like behavior. Further increase to 1 mol % does not lead to qualitative changes. We also point out that Ca^2+^ ions are of low toxicity and the concentrations used during the present polymerization are close to physiological concentrations, to also facilitate suitability for biological applications.

Finally, we address the effect of PIA concentration upon keeping the ratios CXL/IA 4 wt %, GLY 42 wt %, and Ca^2+^/IA 0.5 mol %, as suggested above. Increasing slightly the PIA concentration from 24 wt % (Figure 2g inset) to 27 wt % has a drastic effect of increasing the strength to ∼1000 kPa and of transferring the behavior from soft strain hardening to gross plastic yielding (Figure 2g), still showing a large strain. However, in this case, the 3D-printability by thermal extrusion is not achieved. By contrast, for drastically smaller PIA concentrations, the hydrogel photopolymerization turns inefficient.

The composition of PIA 24 wt %, CXL/IA 4 wt %, GLY 42 wt %, and Ca^2+^/IA 0.5 mol % showed a balance between the strength (75.8 ± 30.0 kPa), strain (1.5 ± 0.4 mm/mm), and processability. Therefore, this composition will be explored subsequently in greater detail. First, the rheological properties to facilitate 3D-printability are explored. Figure 3a shows dynamic rheology using the parallel plate geometry. At room temperature, the storage modulus *Ǵ*∼ 6 × 10^4^ Pa, whereas the loss modulus (*Ǵ*´) is ca. an order of magnitude smaller. Both *Ǵ* and *Ǵ*´ are gradually reduced upon heating, wherein at ca. 64 °C, they cross and *Ǵ*´ becomes larger. This suggests that the compound is a hydrogel at low temperatures and undergoes a smooth transition to a viscous fluid upon heating. This is qualitatively confirmed using the classic vial turning experiment (Figure 3c). To further investigate the melting behavior, differential scanning calorimetry (DSC) was used. Therein, the gel melting is not clearly visible due to its gradual nature (Figure S6). For reference, a similar composition was explored but using a permanent covalent BIS cross-linker instead of CXL (Figure 3b). The parallel plate dynamic rheology showed that *Ǵ* ≫ *Ǵ*´ at all temperatures, as expected of hydrogels with permanent covalent cross-link sites with no bond exchanges.

**Figure 3:**
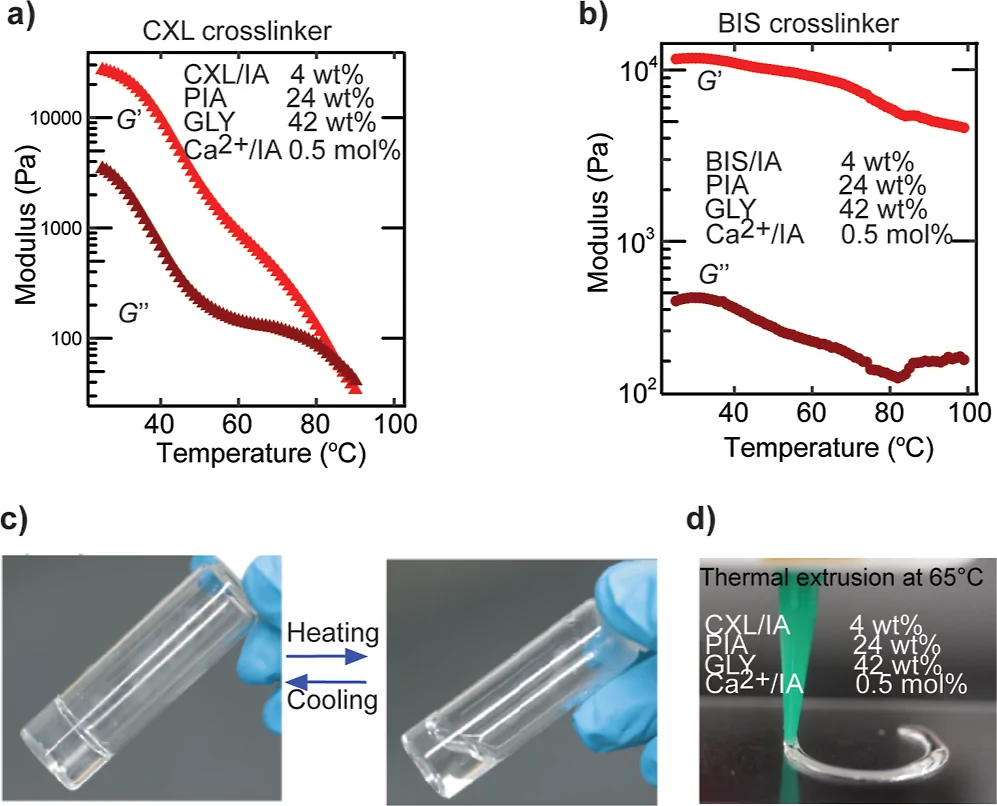
Dynamic rheology and thermally driven gel-to-sol transition, opening the possibility for 3D-printability of the hydrogels. (a) Dynamic plate-and-plate rheology of hydrogels consisting of combined CXL-mediated dynamic adaptable covalent cross-links, Ca^2+^-mediated supramolecular cross-links, and glycerol plasticization. Thermoreversible gelation is inferred. Representative curves shown for 3 parallel samples. (b) A similar composition using a BIS cross-linker does not show gel melting. Representative curves shown for 3 parallel samples. (c) Illustration of thermoreversible flow using the composition of (a). (d) Demonstration of the 3D-printing of a stripe by thermal extrusion at 65 °C followed by fixing by cooling. The composition is PIA 24 wt %, CXL/IA 4 wt %, GLY 42 wt %, and Ca^2+^/IA 0.5 mol %. Samples compared in a measurement have been synthesized independently.

Thus, the gradual softening from hydrogels to viscous liquids points toward the dynamic cross-links provided by CXL. The time–temperature superposition rheology analysis shows that the transesterification process has an activation energy of 44.8 kJ/mol (Figure S10). Large moduli changes upon small-temperature changes and gel-to-sol transition suggests feasibility for low-temperature 3D-printing. Therein, we used a bioprinter (Brinter) to demonstrate the possibility for thermal extrusion printing of the above-mentioned hydrogel. As a proof of concept, stripes could be printed at 65 °C, as quickly stabilized upon natural cooling to room temperature (Figure 3d and Video S1), without needing postprocessing. Additional rheological data relevant for 3D-printing is shown in Figure S8.

The previous discussion already demonstrated that adding a small concentration of divalent Ca^2+^-mediated supramolecular cross-links promote the hydrogel mechanical properties in combination with the dynamic covalent CXL-mediated cross-links. Next, we explore the effects of different metal cations Ca^2+^, Zn^2+^, and Na^+^ and effects of their concentrations in the absence of plasticizing glycerol. Figure 2a already shows that the composition PIA 42 wt %, CXL/IA = 4 wt %, and Ca^2+^/IA 0.5 mol % in the absence of added GLY leads to brittle hydrogels with a strength ∼600 kPa without any yield (duplicated in Figure 4a for clarity). Note that the concentration of Ca^2+^ cannot be drastically increased in this case due precipitation in the reaction mixture. To explore whether a substantially higher concentration of divalent cations could allow promoted mechanical properties, we explored Zn^2+^ which allows dissolution of higher concentrations upon mixing zinc acetate in the polymerization mixture. Figure 4a shows the composition with PIA 36 wt % and CXL/IA = 4 wt % without added GLY, using a high concentration of Zn^2+^/IA 30 mol %. A grossly different behavior with a high strength of ∼800 kPa is observed, showing the onset of yielding. Also, Na^+^ allows high concentration to be used based on the high aq. solubility of NaOH, and therefore even Na^+^/IA 100 mol % could be used in the composition (Figure 4a). However, the noncoordinating behavior of Na^+^ leads to low mechanical properties in this case, as it does not lead to supramolecular cross-linking.

**Figure 4:**
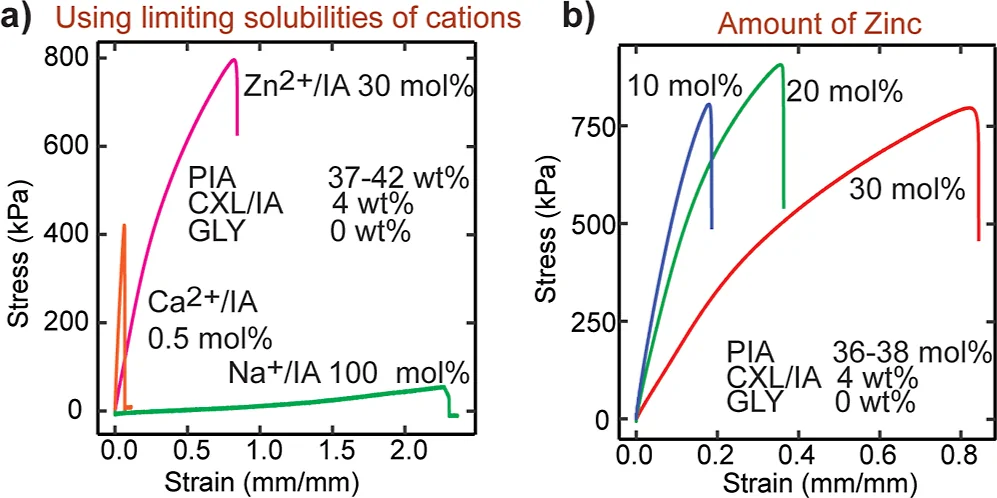
Effects of different metal cations on the hydrogel mechanical properties in the absence of a glycerol plasticizer. (a) The low solubility of Ca^2+^ limits the achievable concentration of the supramolecular cross-links compared to Zn^2+^ and leads to brittleness in the absence of a plasticizer. Zn^2+^ leads to high strength and promotes ductility. Na^+^ allows full neutralization and elastomer-like mechanical properties. (b) Effect of different Zn^2+^ concentrations. Samples containing none of Ca^2+^, Zn^2+^, or GLY plasticizers were too brittle to be measured. Graphs are a representative for at least *n* = 3 number of samples. Samples compared in a measurement have been synthesized independently.

This behavior is discussed in more detail. Na^+^ has only a weak interaction with carboxylate groups. In fact, in water-rich environments, water effectively outcompetes carboxylates in Na^+^ binding sites, leading to nonbridging monodentate interactions between carboxylates and Na^+^,^33^ which in turn allows for a higher ionic strength leading to higher water retention without ion-induced chain stiffening. Ca^2+^ has weak bonding energies with carboxylates, despite its ionic size, as its valence electrons are not in d orbitals.^34^ As a result, there exist bridging supramolecular cross-linking interactions that increase mechanical properties, but they are labile and are easy to break. Later, we show that this, however, allows a thermally driven gel-to-sol transition. Zn^2+^ ions allow stronger coordination bonds, stemming from its higher electron binding affinity and its valence electrons being in d orbitals.^35^

We have specifically chosen Ca^2+^ and Zn^2+^ for their prevalent use in biomedical hydrogel applications.^36^ The Ca^2+^ concentrations are close to physiological conditions. Zn^2+^ is also commonly used in biomedical applications for its antimicrobial properties making it a good candidate for, e.g., infection preventing wound dressings.^37^ In Figure 4b, the effect of different added Zn^2+^ concentrations upon keeping the PIA composition at 36–38 wt %, GLC/IA 4 wt %, and GLY 0 wt % is shown. Therefore, increased weight fraction of Zn^2+^ led to plasticization allowing the onset of yield, wherein plasticizer GLY is not needed. However, unlike the weaker supramolecular interactions of Ca^2+^ that allowed the hydrogel melting already at 65 °C, the stronger interactions with Zn^2+^ prevent the polymer network from melting at such a temperature, prohibiting 3D-printing at low temperatures (Figure S9). Interestingly, the amount of Zn^2+^ dictates the stretchability but not necessarily the ultimate tensile strength of the sample, suggesting that the complex coordination adds plasticity to the network but does not additionally strengthen it. In conclusion, the nature of the interactions of Na^+^, Ca^2+^, and Zn^2+^ with the carboxylate groups in the gel gives the material differing mechanical profiles.

Next, we turn to the aqueous swelling properties of the hydrogels. First, we again discuss the composition of PIA 24 wt %, CXL/IA 4 wt %, GLY 42 wt %, and Ca^2+^/IA 0.5 mol %, which has shown to lead to feasible mechanical and rheological properties, also allowing 3D-printing. Its swelling capacity for Milli-Q water turns to be moderate 188 ± 80 g/g (I, Figure 5a) To improve the swellability and water interactions, we increased the Na^+^ neutralization of IA from 50% to 100% and removed the Ca^2+^-based supramolecular cross-links to reduce cross-linking density. Upon increasing the neutralization degree to 75%, the swelling kept essentially the same, i.e., 175 ± 24 g/g, whereas for the higher neutralization degree of 100% the swelling doubled to 384 ± 92 g/g, presumably due to an increased ionic strength. This absorption value is comparable to those reported by commercial PAA-based superabsorbents^40,41^ (Figure 5a). The shown value shows emerging potential for technical applications as bioderived, environmentally friendly superabsorbents based on biosourced monomers and cross-linkers and sustainable processing. This potential bears high significance, owing to degradation times of 500 years for poly­(acrylic acid) in diapers being a severe issue in the current landscape of microplastics.^38^

**Figure 5:**
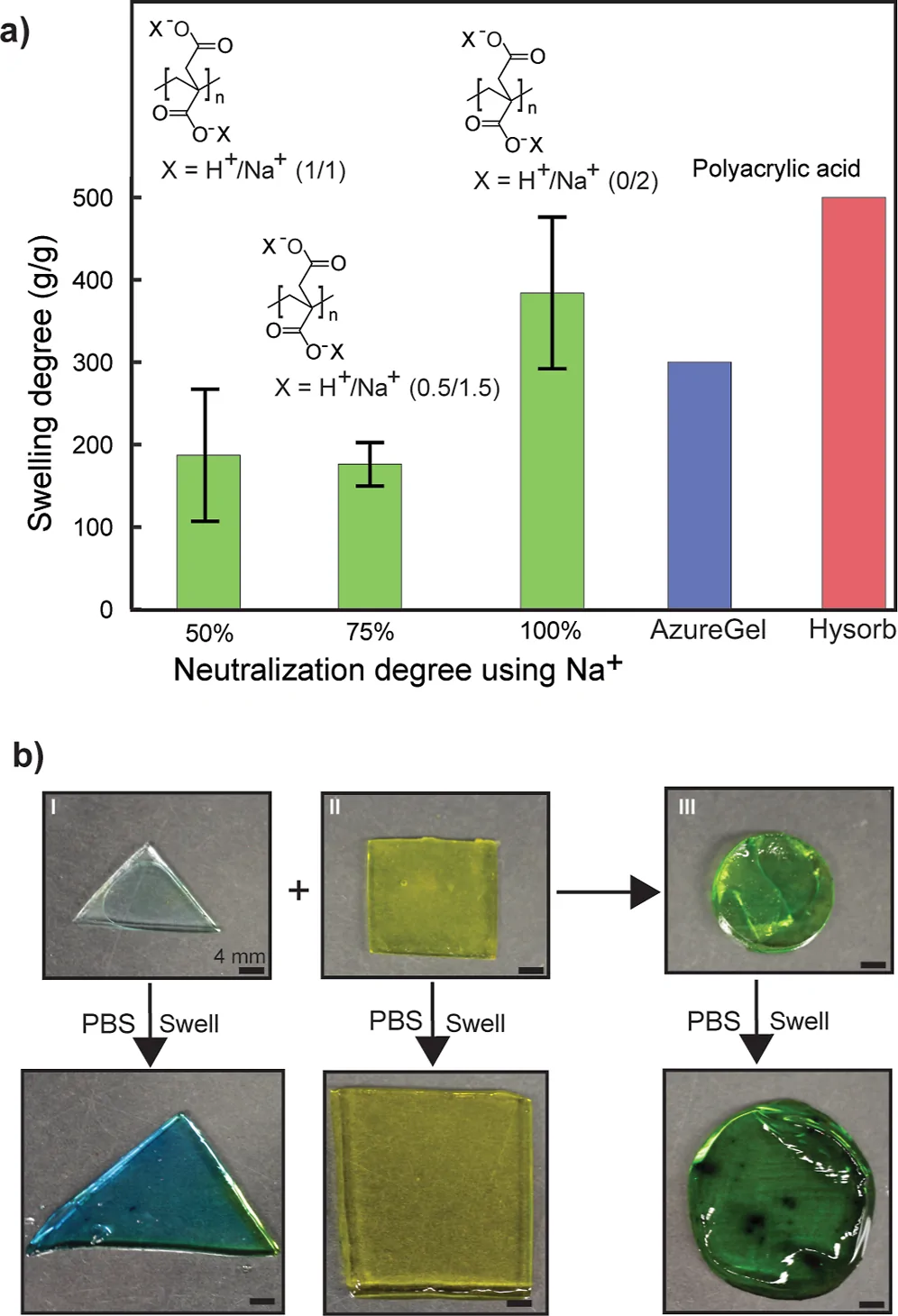
Swelling properties of the polyitaconic acid hydrogels. (a) Aqueous swelling at different levels of Na^+^-neutralization degree in comparison to two commercial poly­(acrylic acid) gels. PIA 37–42 wt %, CXL/IA 4 wt %, and GLY 0 wt %: (b) To visualize dynamic adaptable cross-linking and swelling ability in biological fluids, triangular and rectangular pieces of hydrogel were prepared, both consisting of PIA 24 wt %, 4 wt % CXL vs IA, 43 wt % GLY, and 0.5 mol % Ca^2+^ vs IA. Graphs represent at least *n* = 3 number of samples. Samples compared in a measurement have been synthesized independently.

On the other hand, to explore swelling of biologically relevant compositions, Figure 3d and Video S1 show that the composition 24 wt %, CXL/IA 4 wt %, GLY 42 wt %, and Ca^2+^/IA 0.5 can be 3D-printed at 65 °C by thermal extrusion. Figure 5b (panel I and II) shows that such a composition facilitates swelling also using aqueous Phosphate-Buffered Saline (PBS), to simulate biological conditions. Also, the 3D-printed circular shapes (Figure S8c) can be swollen in PBS without dissolving, though it suffers deformation due to the change of the network cross-linking density and high swelling capacity.^4^ The resulting gel is very soft while water uptake is high. By contrast, the composition prepared without a condensate cross-linker dissolved under the same conditions (Figure S7). This opens the possibility of using this hydrogel as a bioink in the biomedical field such as 3D-printed wound dressings or organ growth scaffolds.^35^

Finally, the swelling of this composition also allows us to illustrate the reversible nature of the covalent and supramolecular cross-links.^44–46^ To demonstrate this dynamic behavior, hydrogel samples have been shaped into a triangular and a square shape (Figure 5b). The triangular sample was dyed blue with methylene blue and rectangular sample yellow by tartrazine for clarity. Both materials undergo separate swelling in aqueous media. Both pieces were heated up to 85 °C for melting, mixing, and molding into a circular sample, and subsequently cooling (Figure 5b III). The resulting gel shows a green color that suggests mixing and reconstitution of the two composing blue and yellow networks, while also showing swelling which shows that, after melting, the resulting gel is cross-linked and insoluble. This is another indirect evidence of the dynamic covalent and supramolecular nature of the gel cross-links of the hydrogel. Hereby, not only the dynamic nature of metal coordination is incorporated but also the above-mentioned reversibility of ester bonds, governed via transesterification reactions.^39^ The network contains covalent ester groups at the cross-linker and provides abundant carboxyl groups throughout the hydrogel. That the covalent cross-links are relevant to stabilize the hydrogel is demonstrated in Figure S7 which shows that omission of CXL leads to quick dissolution.

## Conclusion

In conclusion, in this work we demonstrate the potential of PIA as a convincing alternative to fossil fuel-based polyacrylates. The herein synthesized compounds showed properties similar to established PAAs. The concept is based on a combination of dynamic covalent and supramolecular cross-links. The covalent cross-linker is a condensation product of esterification between glycerol and itaconic acid, synthesized in the absence of added solvents without purification steps to promote sustainability. The different reaction products in the glycerol–itaconic acid condensate synthesis contribute to provide dynamic covalent cross-links for the hydrogel. The supramolecular cross-links are provided by metal cations interacting with carboxylates. The hydrogels are directly photopolymerized using mixtures of the half-neutralized itaconic acid monomer, the covalent and supramolecular cross-linkers, and added glycerol, using the polymerization photoinitiator α-ketoglutaric acid, also which can be biosourced. Upon selecting the compositions, the hydrogel mechanical properties are highly tunable, from a high strength of ∼1000 kPa involving plastic yielding to softer materials with elastomer-like strain hardening behavior. When using Ca^2+^ as the supramolecular cross-linker at near-physiological concentrations, the hydrogel can be melted upon slight heating to 65 °C, thus allowing for reprocessability despite the chemical cross-links. This is possible owing to dynamically exchangeable transesterification reactions being promoted at slightly elevated temperatures. Furthermore, we show 3D printing via thermal extrusion to be a viable processing option. The 3D-printed shapes could be swollen in aqueous Phosphate-Buffered Saline (PBS) without dissolving, which opens the possibility for biomedical applications such as wound patches or growth scaffolds. As the material is mechanically stable and incorporates biocompatible components, the hydrogel films for target drug delivery and cell scaffold can suggest potential biomedical applications even if not directly assessed in this work. Use of Zn^2+^ instead of Ca^2+^ as the supramolecular cross-linkers increases the toughness of the network significantly based on a stronger coordination thus allowing for, e.g., soft robotics applications. Increasing the neutralization degree from 50% to 100% using Na^+^ results in a biobased superabsorbent with a swelling degree of 384 + – 92 g/g. Thus, we show that the poly­(itaconic acid) hydrogel allows a flexible and versatile platform toward sustainability for different functions which can be tuned for a variety of applications via the present hybrid dynamic covalent-supramolecular gel network approach.

## Supplementary Material





## Notes

Experimental section and methods; condensate cross-linker CXL characterization by ESI-QTOF and H1-NMR; and poly­(itaconic acid) hydrogel properties (PDF)
Video showing the thermal extrusion of a cross-linked polyitaconic acid gel (MP4)
