# Supplementary material for: Poly(itaconic Acid) Hydrogels with Dynamic Covalent and Supramolecular Cross-Links for High and Tunable Mechanical Properties
Source: ACS Appl Polym Mater. 2026 Jul 22;8(15):12367–75. doi: 10.1021/acsapm.6c01440 (PMC13488272; doi:10.1021/acsapm.6c01440)
Supplement: Supplementary file 1 [file ap6c01440_si_001.pdf]

Supporting Information

Poly(itaconic acid) hydrogels with dynamic covalent and supramolecular cross-links for high and tuneable mechanical properties

Henri Savolainen[1], Maximilian J. L. Hagemann [1], Mika Salmi[2], Jouni Partanen[2], Mario Piedrahita-Bello[1], Olli Ikkala[1*]*

[1]Department of Applied Physics, Aalto University, 02150 Espoo, Finland

[2]Department of Energy and Mechanical Engineering, Aalto University, 02150 Espoo, Finland

Email: mario.piedrahitabello@aalto.fi; olli.ikkala@aalto.fi

1. Experimental Section and Methods

Materials: Itaconic acid (IA), sodium hydroxide, calcium chloride, glycerol, 2-oxoglutaric acid, iron (III) chloride, zinc acetate dihydrate, *N,N'*-methylenebisacrylamide (BIS), methanesulfonic acid (MSA), ammonium persulfate, tetramethylethylendiamine, and Irgacure 2959 were purchased from Sigma Aldrich and used without any further purification.

Synthesis condensate crosslinker (CXL): Itaconic acid (39.0 g, 0.300 mol, 3.0 eq.) and glycerol (9.2 g, 0.100 mol, 1.0 eq.) were stirred at 170 °C for 10 minutes without using additional solvents. The mixture was cooled to 120 °C and the catalyst MSA (0.04 g, 0,41 mmol, 0,004 eq.) was added, as well as hydroquinone as an inhibitor for protecting the vinyl groups, and the mixture was stirred at room temperature for 1.5 hours. The resulting yellow solution was cooled to 7 °C overnight to yield the crosslinker (CXL) as a viscous paste. We emphasize that to promote sustainable synthesis, no additional solvents or purification steps were involved.

Neutralization of the itaconic acid monomer: Itaconic acid (10.0 g, 0.077 mmol, 1.0 eq.) was dissolved in 10 mL Milli-Q water and sodium hydroxide (3.07 g, 0.077 mmol, 1.0 eq.) was added at 0 °C under constant stirring to prepare stock solution 1. Weight percentages in the text are given as ratios of monomer to water during the stock solution mixing.

Synthesis poly(itaconic acid) hydrogel: An example to synthesize the composition PIA 24 wt%, 4 wt% CLX *vs.* IA, 43 wt% GLY, and 0.5 mol% Ca^{2+} vs IA: 2-oxoglutaric acid (0.015 g, 0.0001 mmol, 0.001 eq.), the abovementioned stock solution (1 mL), CXL (0.03 g), calcium chloride

aqueous solution (10 µL 200 mg/mL) and glycerol (1.00 g) were quickly vortexed at room temperature. The reaction mixture was sonicated until full dissolution of the crosslinker was observed and the solution was injected into a glass mold, transferred to a UV chamber (8 lamps of 14 W, 350 nm) and irradiated for 2 hours to obtain a bulk sample.

Tensile tests: The tensile tests were measured on an Instron 68TM-5. The samples were stamped to a dumbbell shape with a width of 2 mm. The samples were clamped onto the grippers with 15 mm distance. The extension speed of the tensile tests was 1 (mm/mm) min^{-1}.

Rheology: The samples were synthesized in a mold of 0.5 mm thickness. They were tested under 1 % strain and a frequency of 1 Hz. Temperature ramps of 25 - 70 and 25 - 99 °C were performed in 1 °C/min steps. For yielding and flow tests, samples were heated to a given temperature and then let equilibrate for 15 minutes.

3D printing demonstration: A ring-shaped sample was designed in Solidworks and printed with a Brinter 3D bioprinter using a batch of the aforementioned hydrogel. The ink was held at 65 °C for 1 hour prior to printing to ensure complete liquification. Samples were printed at 75 kPa at a speed of 5 mm/s.

Swelling studies: The samples for the swelling studies were irradiated during synthesis for 6 hours instead of 2 hours. Samples were swollen in Milli-Q water overnight and then dried. Dried samples were swollen again in Milli-Q water and the swelling ratio was determined by the increased weight.

$$Q = \frac{g_{swollen} - g_{dry}}{g_{dry}}$$

Neo-hookean fit: Tensile test samples have been fitted from 1 to 1.2 strain with the neo-hookean equation and then from there the effective crosslinking density was calculated.

$$\sigma = G(\lambda - \lambda^{-2})$$

$$v = \frac{G}{RT}$$

Activation energy: The activation energy was done via rheological measurements with 2.5°C steps. From the horizontal shifts, the activation energy was determined via linear fit.

$$\ln(\alpha) = \frac{E}{2.03R}\left(\frac{1}{T} - \frac{1}{T_{Ref}}\right)$$

2. Condensate Cross-Linker CXL Characterization by ESI-QTOF and H1-NMR

The ESI-QTOF and H1 NMR characterization of the reaction products between IA and GLY to synthesize CXL is presented in **Figures S1-S4,** also showing the reaction products.

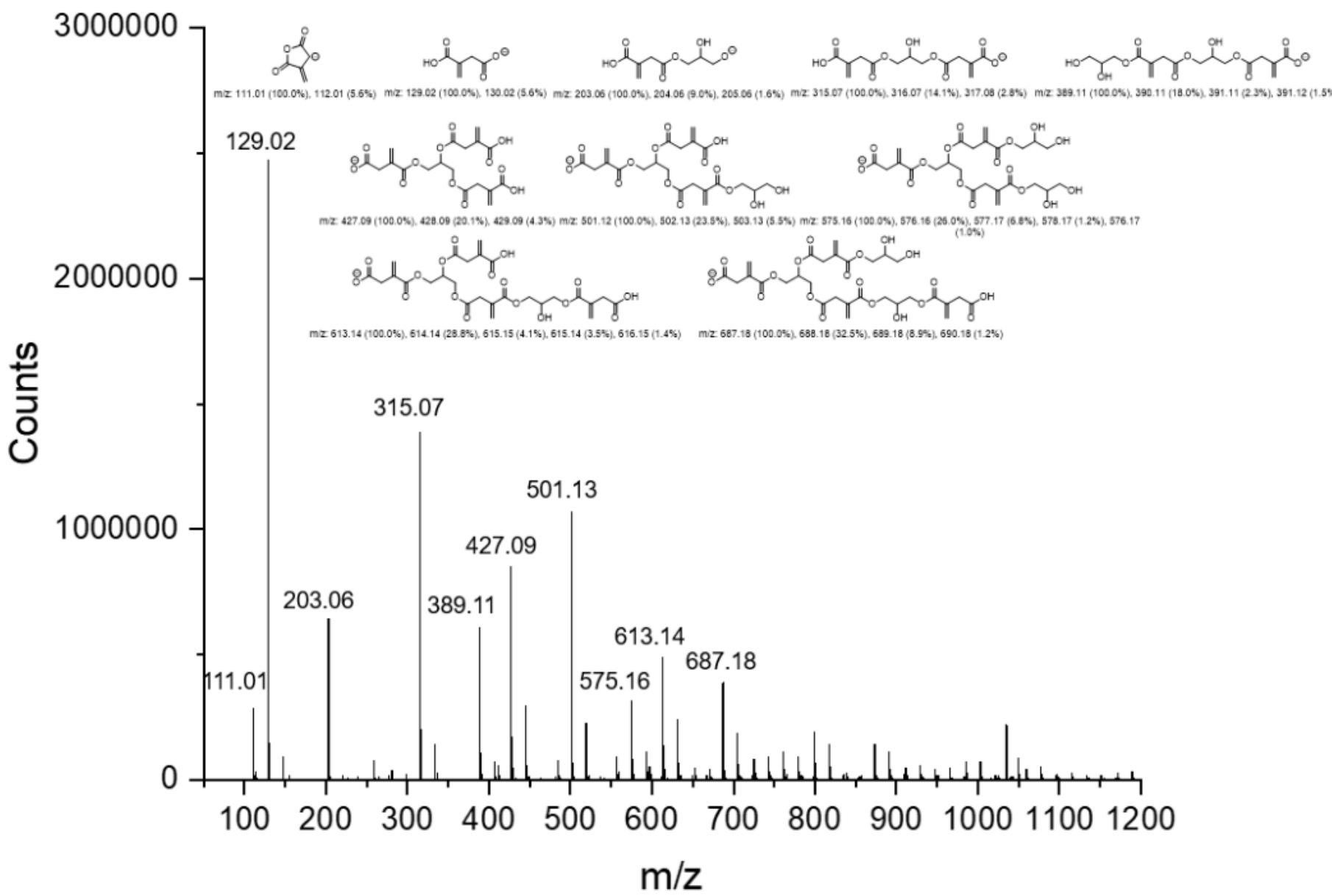

Figure S1. ESI-QTOF of CXL. The structures of the 10 highest peaks are displayed and marked.

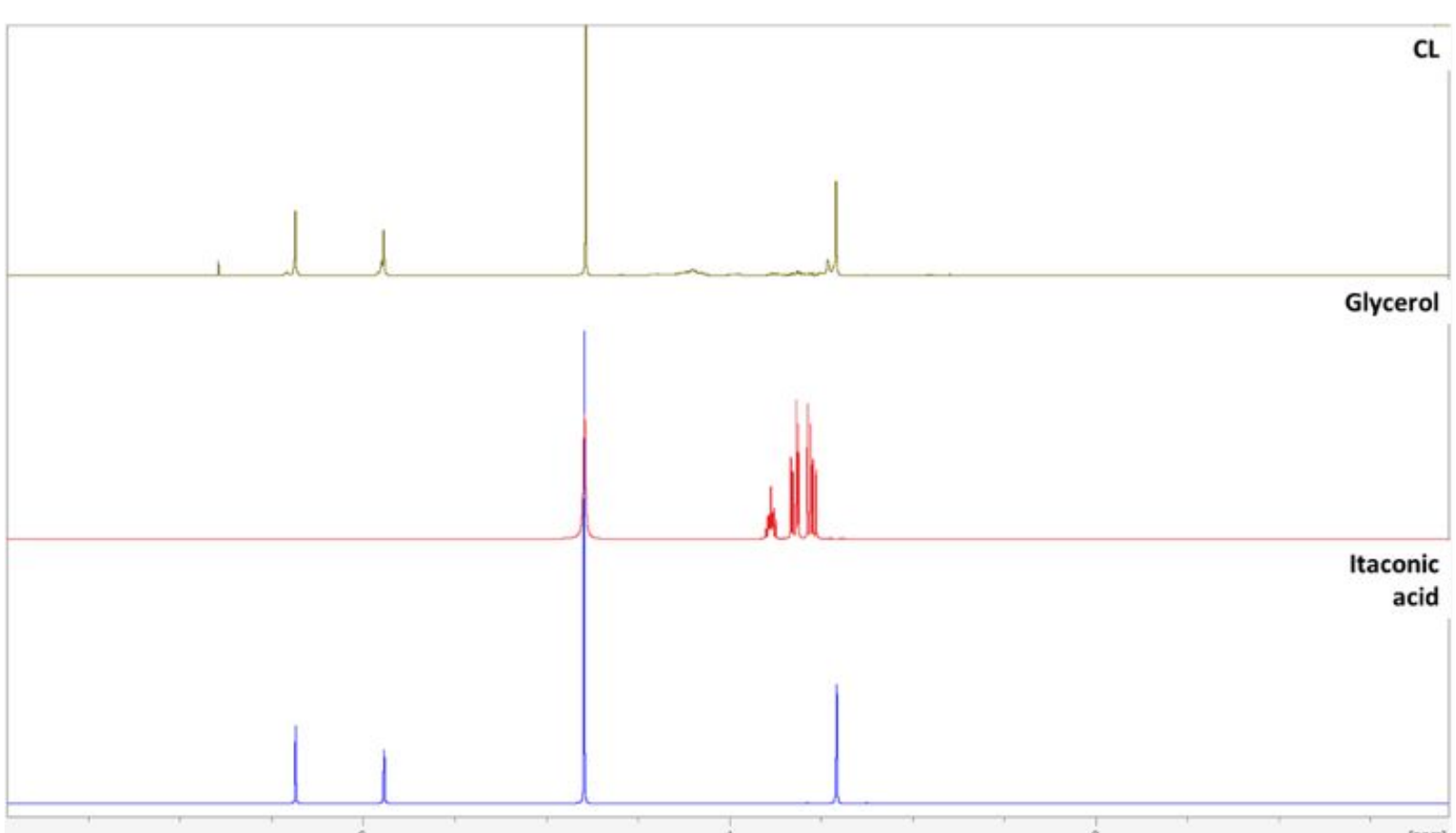

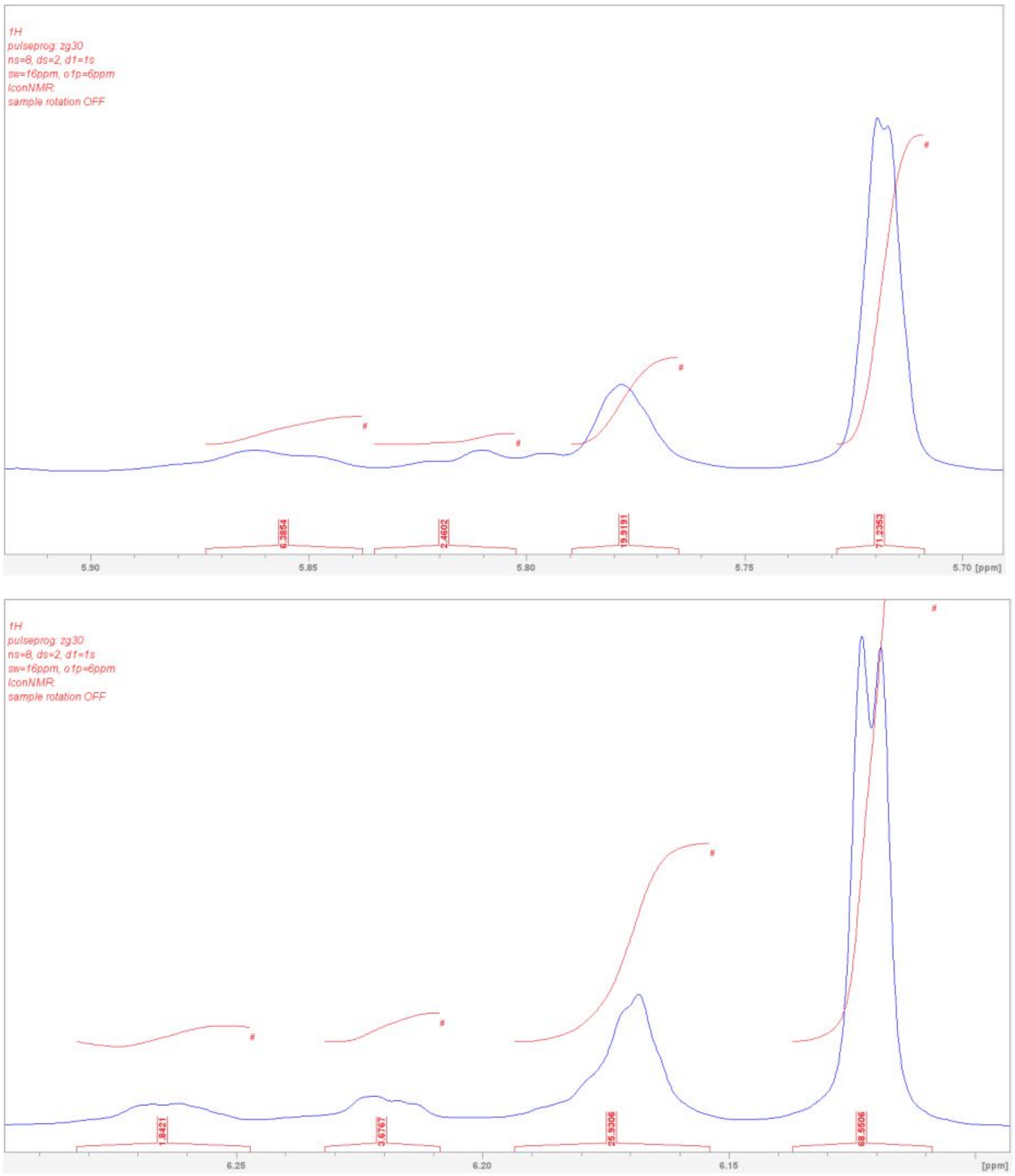

Figure S2. 1H NMR of the CXL condensate and reagents in D_2O.

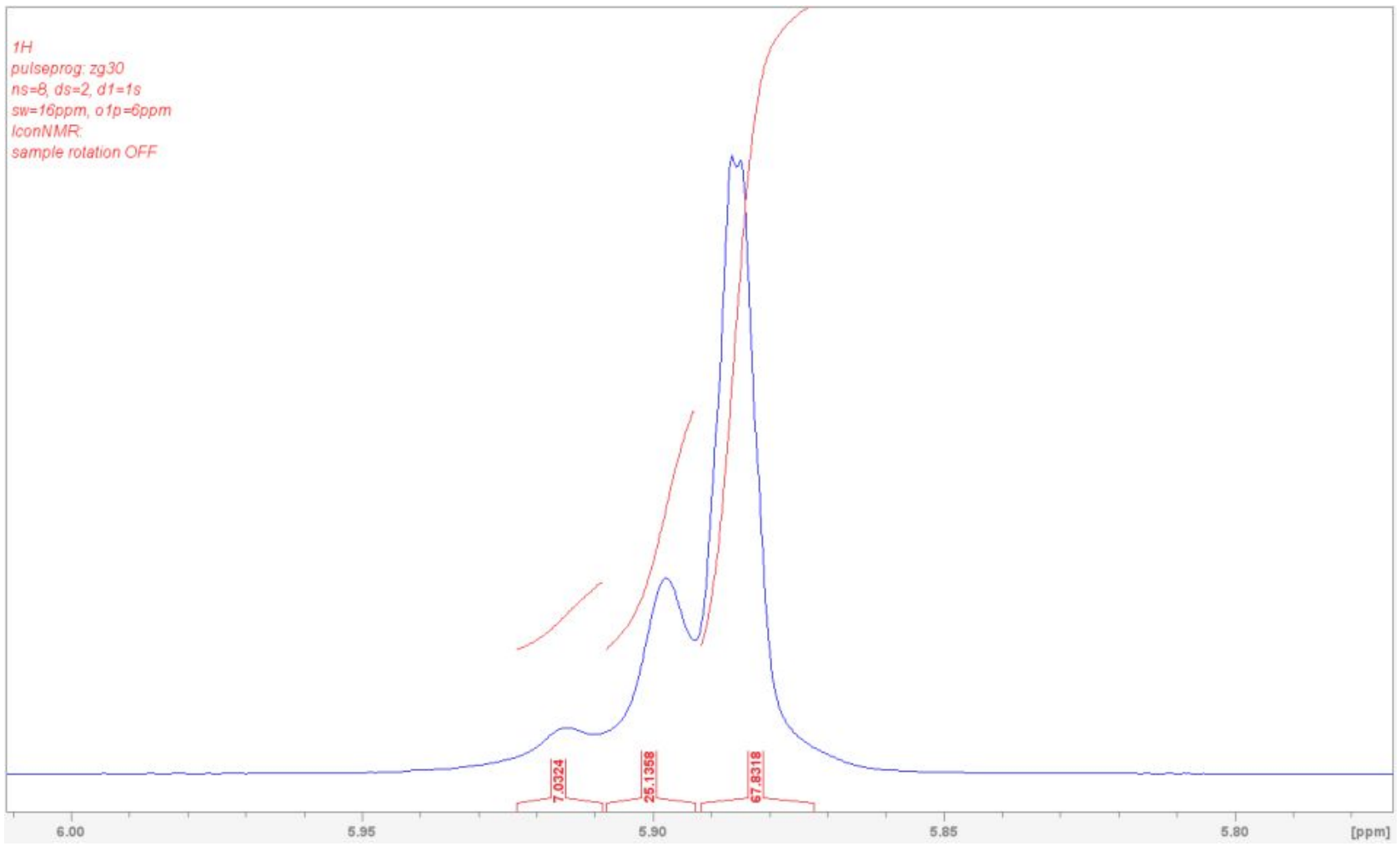

Figure S3. 1H NMR of the CXL condensate and reagents in D_2O: Partial 1H NMR spectrum of the alkene proton peak at 5.87 ppm, showing the integrals of the unreacted itaconic acid (left; 68%) and the condensated itaconic acid (other two peaks; 32%).

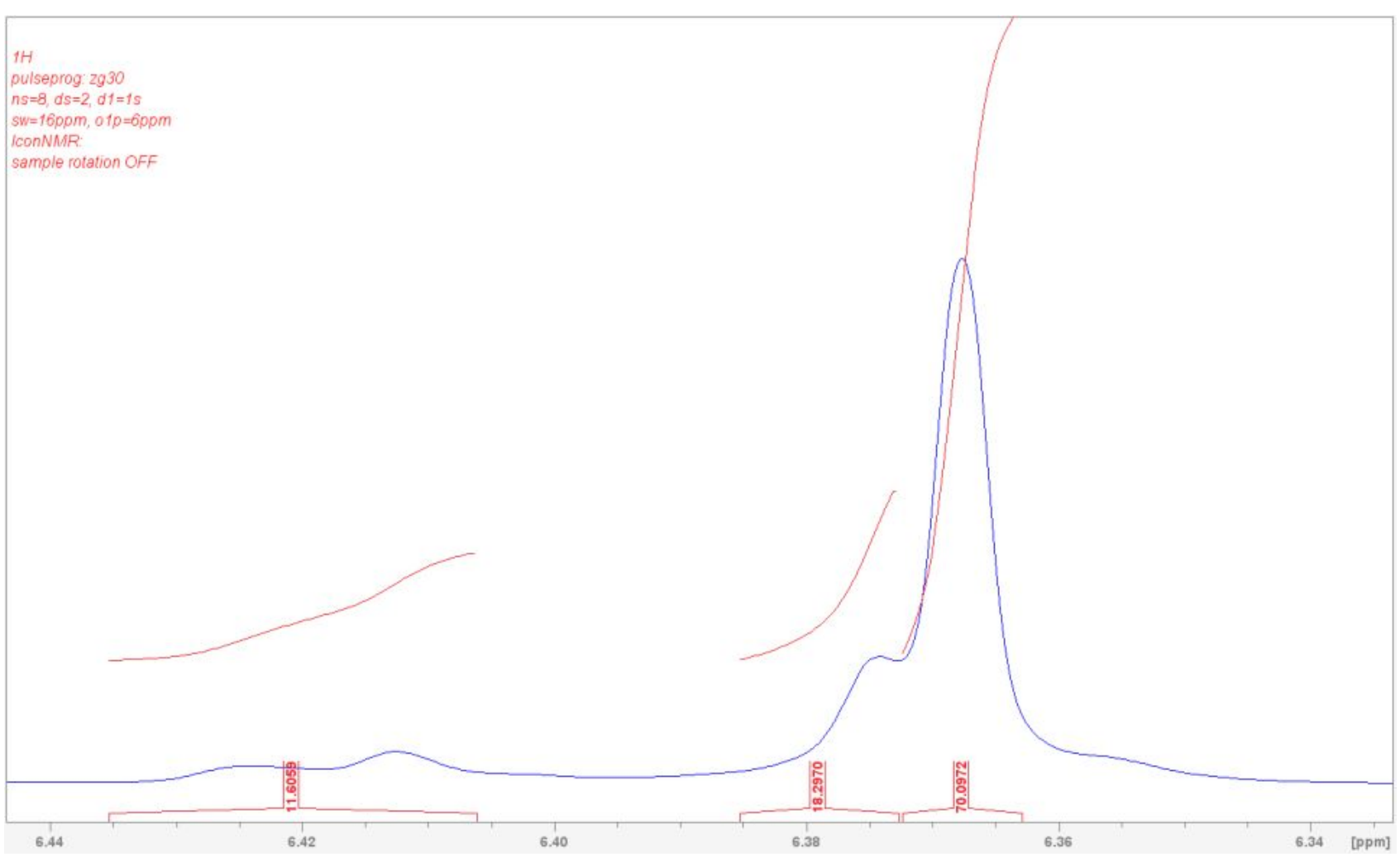

Figure S4. H1 NMR of condensate CXL and reagents in D_2O: Partial 1H-NMR spectrum of the alkene proton peak at 6,37 ppm, showing the integrals of the unreacted itaconic acid (left; 70%) and the condensated itaconic acid (other two peaks; 30%).

3. Poly(itaconic acid) Hydrogel Compounds

Table S1 lists the compounds synthesized and characterized. The cross-linker is CXL, unless specified to be BIS.

Figure	Curve Color	IA(wt%)	CXL/IA (wt%)	Gly (wt%)	Cation 1 Neutralization	mol% vs. IA	Cation2 Cross-linking	mol% vs. IA
2a	Blue	42	4,1	0	Na	50	Ca	0,5
2a	Green	30	4,1	27	Na	50	Ca	0,5
2a	Red	24	4,1	42	Na	50	Ca	0,5
2b	Blue	24	Bis 4,1	42	Na	50	Ca	0,5
2b	Red	24	4,1	42	Na	50	Ca	0,5
2c	Blue	24	4,1	42	Na	50	Ca	0,5
2c	Orange	24	4,1	42	Na	50	Ca	0,5
2e	Red	24	6,2	43	Na	50	Ca	0,5
2e	Orange	24	4,1	42	Na	50	Ca	0,5
2e	Black	24	8	42	Na	50	Ca	0,5
2f	Blue	24	4,1	43	Na	50	Ca	0,25
2f	Red	24	4,1	42	Na	50	Ca	0,5
2f	Green	24	4,1	42	Na	50	Ca	1
2g	Red	24	4,1	42	Na	50	Ca	0,5
2g	Blue	27	3,7	42	Na	50	Ca	0,44
3a	Red	24	4,1	42	Na	50	Ca	0,5
3b	Red	24	Bis 4,1	42	Na	50	Ca	0,5
3c	Red	24	4,1	42	Na	50	Ca	0,5
3d	Red	24	4,1	42	Na	50	Ca	0,5
4a	Orange	42	4,1	0	Na	50	Ca	0,5
4a	Pink	36	4,1	0	Na	50	Zn	30
4a	Green	37	4,6	0	Na	100	-	-
4b	Blue	40	4,1	0	Na	50	Zn	10
4b	Green	38	4,1	0	Na	50	Zn	20
4b	Red	36	4,1	0	Na	50	Zn	30
5a	Green	42	4,1	0	Na	50	-	-
5b	Green	39	4,3	0	Na	75	-	-
5c	Green	37	4,6	0	Na	100	-	-
5b	-	24	4,1	42	Na	50	Ca	0,5
S5c	Blue	24	2,7	43	Na	50	Ca	0,5
S5c	Red	27	3,7	42	Na	50	Ca	0,44
S6a	Blue	24	4,1	42	Na	50	Ca	0,5
S6a	Red	24	0	43	Na	50	Ca	0,5
S6b	Blue	24	4,1	42	Na	50	Ca	0,5
S6b	Red	24	0	43	Na	50	Ca	0,5
S7	Red	24	0	43	Na	50	Ca	0,5
S8a	Purple	24	4,1	42	Na	50	Ca	0,5
S8b		24	4,1	42	Na	50	Ca	0,5
S8c		24	4,1	42	Na	50	Ca	0,5
S8d	Red	24	4,1	42	Na	50	Ca	0,5
S9	Red	36	4,1	0	Na	50	Zn	30

An example of the sample preparation

As an example of the sample preparation, the synthesis steps for the composition denoted as PIA weight fraction 24 wt%, CXL/IA 4 wt%, GLY 42 wt%, and 0.5 mol% $Ca^{2+/}$IA is shown:

Step 1: Itaconic acid (10.0 g, 0.077 mmol, 1.0 eq.) was dissolved in 10 mL milli-Q water and sodium hydroxide (3.07 g, 0.077 mmol, 1.0 eq.) was added at 0 ^{o}C under constant stirring to prepare stock solution

Step 2: To 0.015 g of 2-oxoglutaric acid and 0.020 g was added 1 mL (so 1.3 g) of stock solution from step 1.

Step 3: To this mixture add 1 g of glycerol.

Step 4: Then, 10 uL of aqueous calcium chloride solution (concentration 200 mg/mL) was added to the solution and vortexed until fully dissolved.

4. Supporting Figures

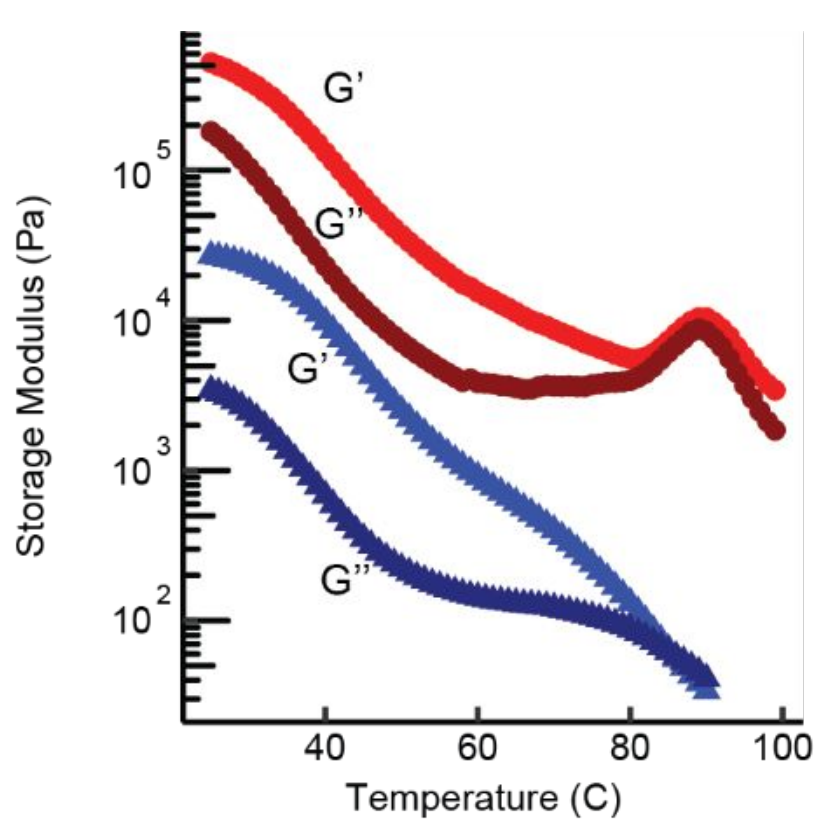

Figure S5. Rheological characterization of the hydrogels at two polymer concentrations. Graphs represent the average of at least n=3 number of samples for the blue curve and n=1 for the red curve. Samples compared in a measurement have been synthesized independently. Average of a within one composition has been taken from different batches.

:

Red curves: Composition: PIA 27 wt%, CXL/IA 3.7 wt%, GLY 42 wt%, and 0.5 mol% Ca^{2+}/IA.

Blue curves: Composition: PIA 24 wt%, CXL/IA 4 wt%, GLY 42 wt%, and 0.5 mol% Ca^{2+}/IA.

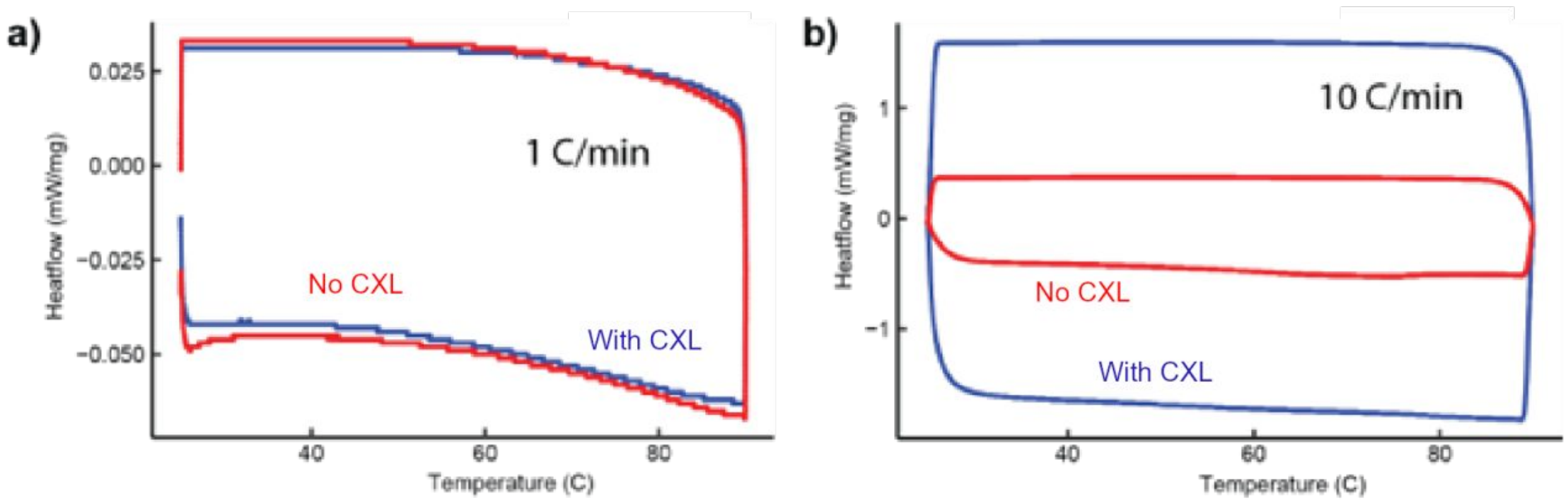

Figure S6. DSC characterization of composition PIA 24 wt%, CXL/IA 4 wt%, GLY 42 wt%, and 0.5 mol% Ca^{2+}/IA. **a)** Low heating rate. **b**) Characterization of samples with and without CXL crosslinker at high heating rate. Graphs represent at least n=1 number of samples. Same sample has been used for the measurement.

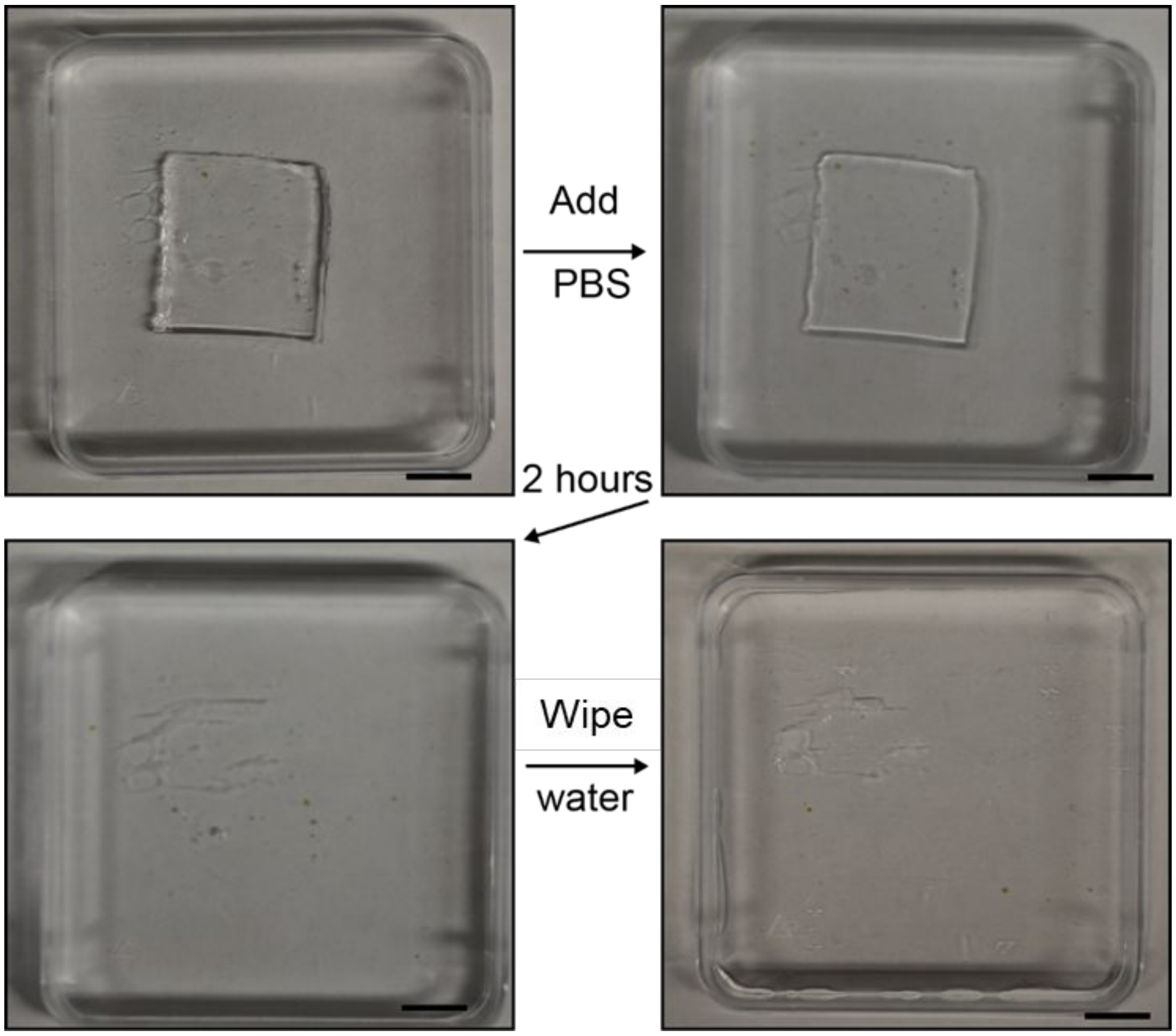

Figure S7. Demonstration of dissolution of non-crosslinked sample of composition: PIA 24 wt%, CXL/IA 0 wt%, GLY 42 wt%, and 0.5 mol% Ca^{2+}/IA. Phosphate-buffered saline (PBS) was used to dissolve the gel. The gel is gently wiped with water to verify dissolution.

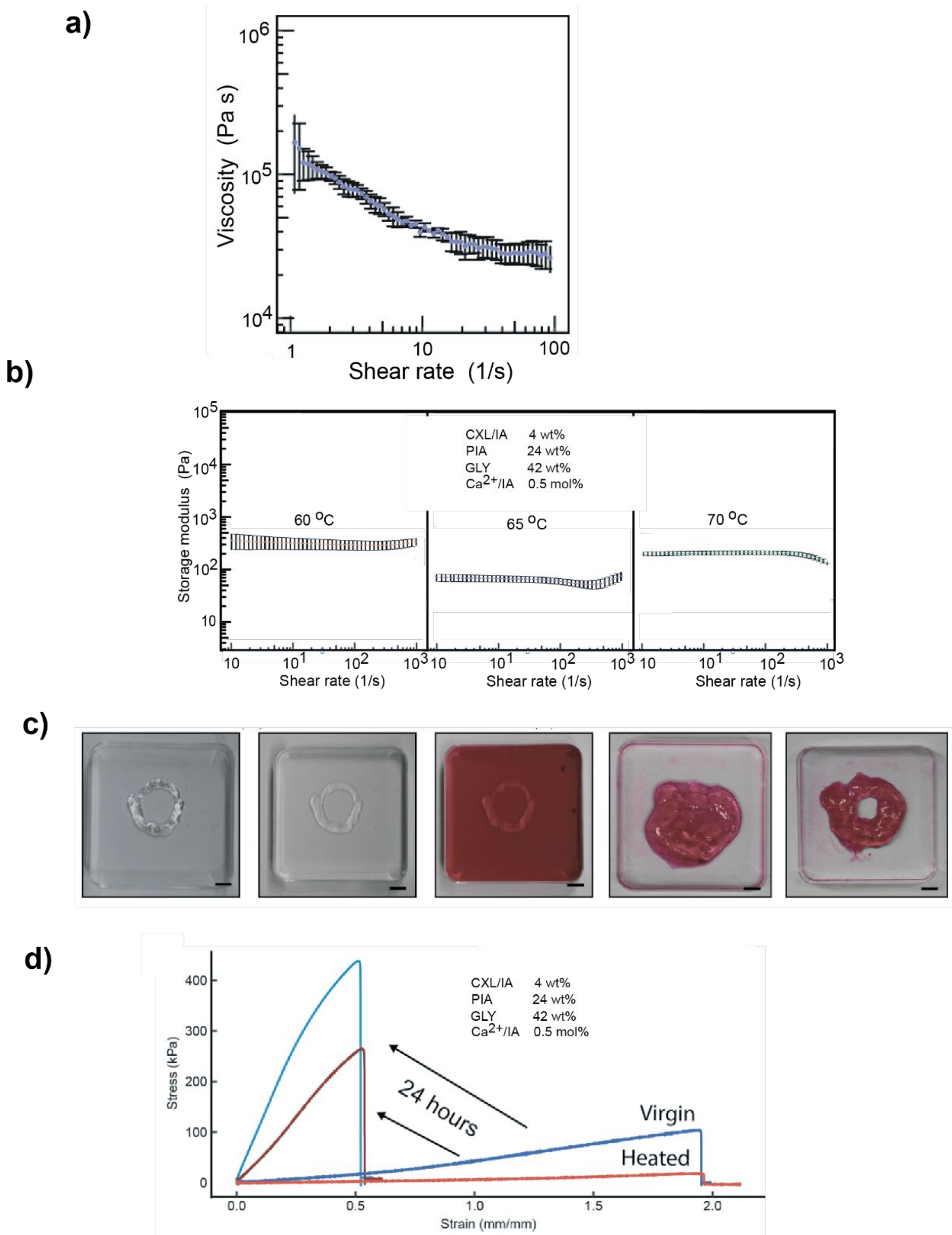

Figure S8. Rheological characterization of composition: PIA 24 wt%, CXL/IA 4 wt%, GLY 42 wt%, and 0.5 mol% Ca^{2+}/IA. **a)** Shear rate sweep. **b)** Stress sweep. **c)** Demonstration of printed ring, swelling in PBS and fuchsin acid. **d**) Comparison of sample that has been heated at 70 ^{o}C for 10 minutes and virgin sample. The second measurement was done after 24 hours, showing qualitatively the reformation of the gel. Graphs represent at least n=3 number of samples. Samples compared in a measurement have been synthesized independently. Average of a within one composition has been taken from one batch.

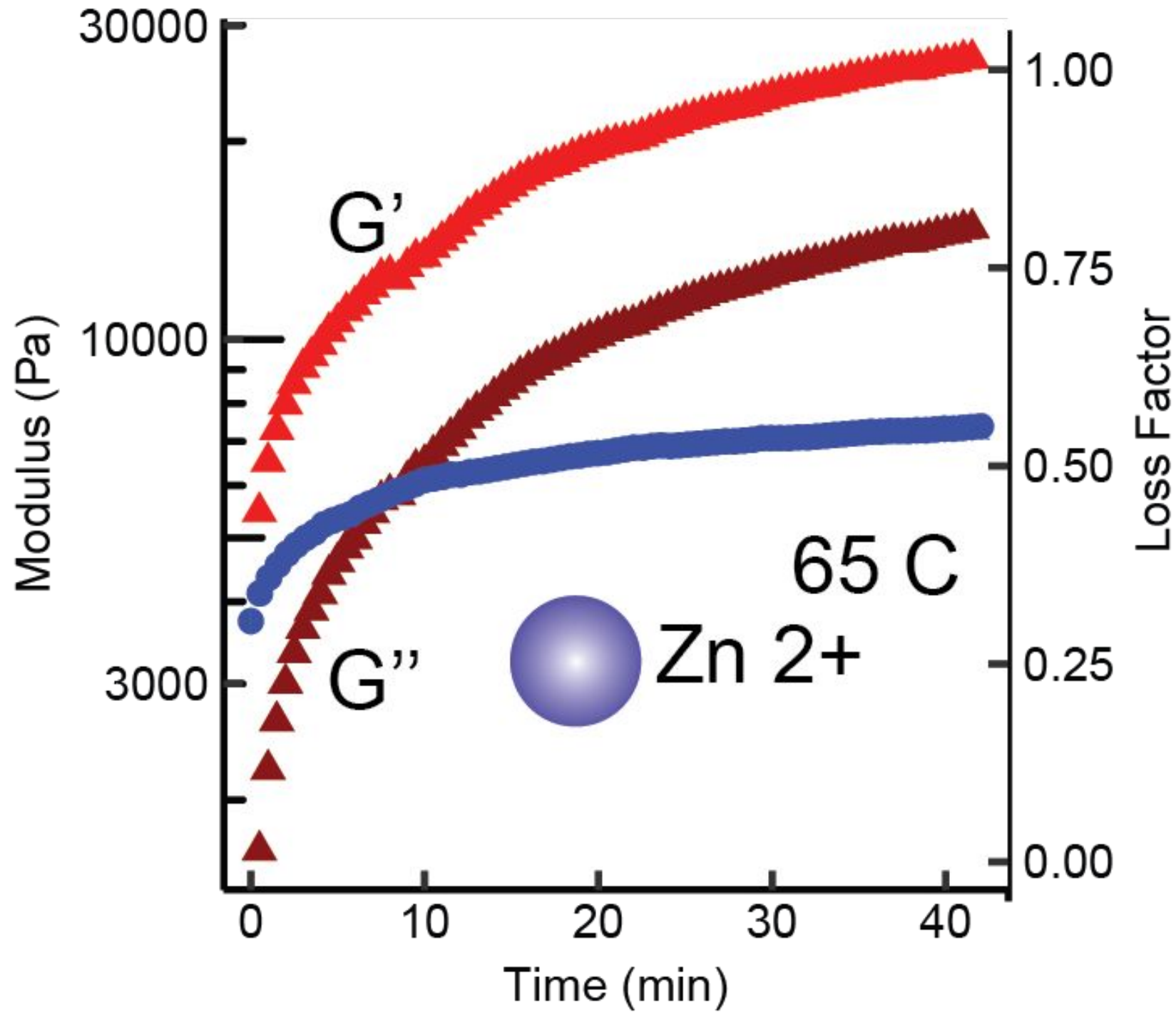

Figure S9. Rheological characterization of the the composition: PIA 36 wt%, CXL/IA 4 wt%, GLY 42 wt%, 30mol%, Zn^{2+} at 65 ^{o}C. Graph represents n=1 number of samples.

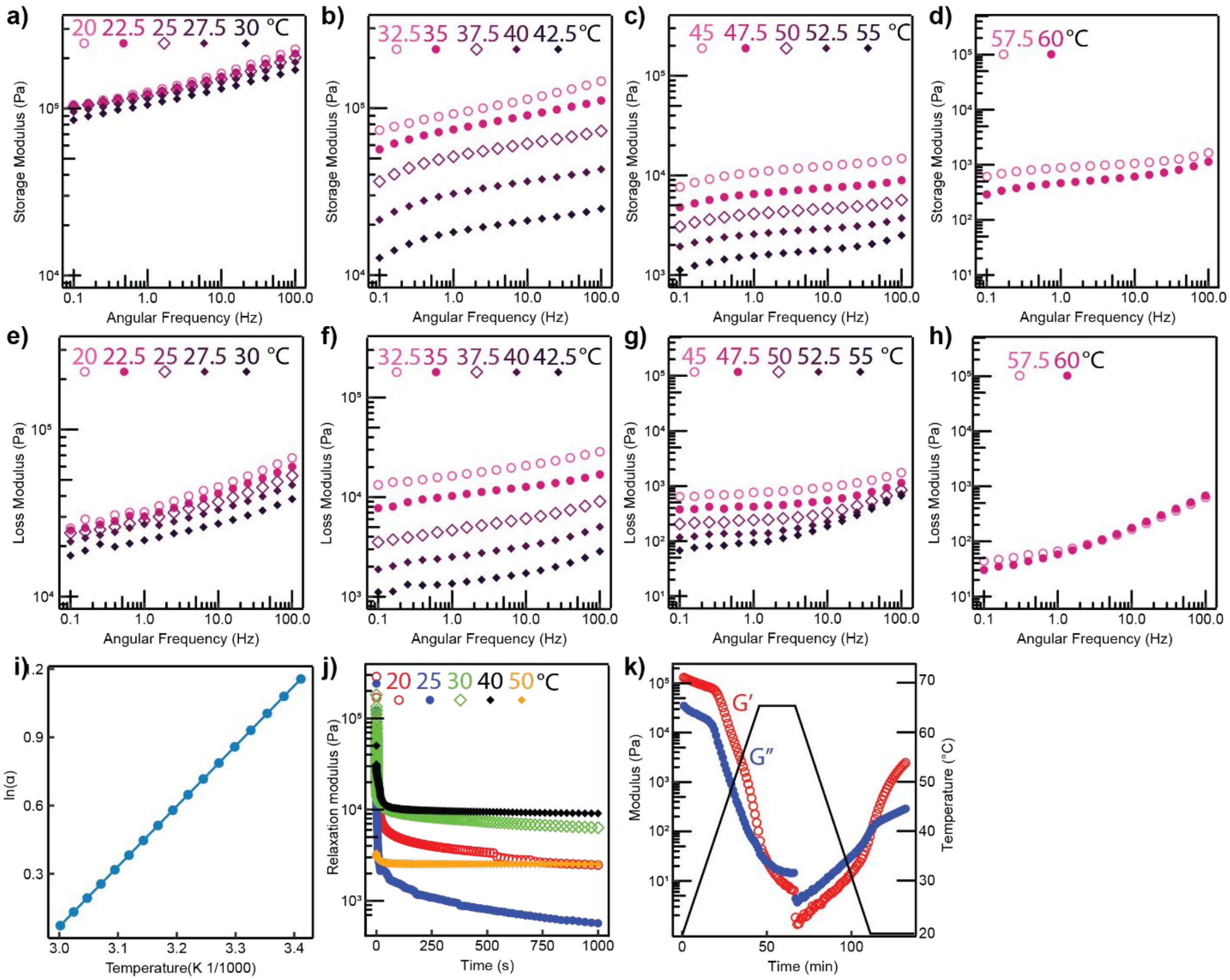

Figure S10: Rheological characterization of dynamic covalent behavior for a sample prepared as PIA 24 wt%, CXL/IA 4 wt%, GLY 42 wt%, and Ca^{2+}/IA 0.5 mol%. a-h) Storage and loss modulus characterization from 20-60°C in steps of 2.5°C. i) Arrhenius plot of shift factor over temperature to determine activation energy. j) Step-strain tests at different temperatures. k) Heat and cooling cycling test from 20 to 65°C. All measurements have been made with n=1 number of samples.
